# MALDI Mass Spectrometry Imaging for the Distinction of Adenocarcinomas of the Pancreas and Biliary Tree

**DOI:** 10.3390/molecules27113464

**Published:** 2022-05-27

**Authors:** Christine Bollwein, Juliana Pereira Lopes Gonҫalves, Kirsten Utpatel, Wilko Weichert, Kristina Schwamborn

**Affiliations:** 1 Institute of Pathology, School of Medicine, Technical University of Munich, Trogerstraße 18, 81675 Munich, Germany; juliana.goncalves@tum.de (J.P.L.G.); wilko.weichert@tum.de (W.W.); kchwamborn@tum.de (K.S.); 2 Institute of Pathology, University of Regensburg, Franz-Josef-Strauß-Allee 11, 93053 Regensburg, Germany; kirsten.utpatel@klinik.uni-regensburg.de

**Keywords:** MALDI-MSI, tandem mass spectrometry, pancreatic ductal adenocarcinoma, cholangiocarcinoma, machine learning, supervised classification, proteomics, peptides

## Abstract

Pancreatic ductal adenocarcinoma and cholangiocarcinoma constitute two aggressive tumor types that originate from the epithelial lining of the excretory ducts of the pancreatobiliary tract. Given their close histomorphological resemblance, a correct diagnosis can be challenging and almost impossible without clinical information. In this study, we investigated whether mass spectrometric peptide features could be employed to distinguish pancreatic ductal adenocarcinoma from cholangiocarcinoma. Three tissue microarrays of formalin-fixed and paraffin-embedded material (FFPE) comprising 41 cases of pancreatic ductal adenocarcinoma and 41 cases of cholangiocarcinoma were analyzed by matrix-assisted laser desorption/ionization mass spectrometry imaging (MALDI-MSI). The derived peptide features and respective intensities were used to build different supervised classification algorithms: gradient boosting (GB), support vector machine (SVM), and k-nearest neighbors (KNN). On a pixel-by-pixel level, a classification accuracy of up to 95% could be achieved. The tentative identification of discriminative tryptic peptide signatures revealed proteins that are involved in the epigenetic regulation of the genome and tumor microenvironment. Despite their histomorphological similarities, mass spectrometry imaging represents an efficient and reliable approach for the distinction of PDAC from CC, offering a promising complementary or alternative approach to the existing tools used in diagnostics such as immunohistochemistry.

## 1. Introduction

Ductal adenocarcinoma of the pancreas (PDAC) and cholangiocarcinoma (CC) represent two malignant tumor entities arising from the epithelium of the pancreatobiliary tree [1,2,3]. Despite their relatively low overall incidence rates both tumor types typically display aggressive growth patterns rendering PDAC and CC the 5th and 16th causes of cancer death worldwide, respectively [4]. Distinction between these two entities on purely histopathological grounds e.g., on a biopsy of a mass lesion of the liver or pancreatic head can be challenging or even impossible given the virtually identical histological phenotype. This, however, has important therapeutic implications regarding options in surgical management and systemic treatment. Moreover, it has a profound impact on the assessment of prognosis [5,6]. A correct assignment can mostly be achieved by an integration of clinical and radiological information, but occasionally equivocal cases remain that do not provide sufficient clues for clear-cut classification [7]. In recent years several attempts have been made to establish new biomarkers (single or panel) that would increase the diagnostic performance in discriminating adenocarcinoma of the biliary system from ductal adenocarcinoma of the pancreas. Biomarker discovery, however, is hampered by intra- and intertumoral heterogeneity as shown by proteomic and molecular studies [8,9,10].

Matrix-assisted laser desorption ionization-mass spectrometry imaging (MALDI-MSI) is an analytical technique that enables the assessment of the spatial distribution and relative abundance of molecular classes such as peptides and proteins in intact tissue sections [11]. Without the need for target-specific reagents, such as antibodies, several hundreds of analytes can be measured in parallel [12]. The spectrum obtained for every scanned pixel represents a plot of *m/z* ions (mass to charge ratios) against their relative intensities, with a single or limited number of molecules underlying a specific *m/z* value. For each analyte, an ion density map can be generated and visualized in projection on the analyzed tissue section. In addition, the very same section can be subsequently stained with hematoxylin and eosin (H&E) or immunohistochemically (IHC) to correlate histological findings with the relative abundance of different analytes [13]. This technology has already proven powerful in distinguishing different types of cancers in tissue microarrays [14,15,16].

In this pilot study, we have used MALDI-MSI for the investigation of relevant proteomic features that can aid in the diagnosis of PDAC and CC. The different spectra recorded were also used to train and test supervised classification models, which could be used to assist the current pathological analysis of the tissue. This process would enable a faster diagnosis and a better selection of the treatment course.

## 2. Materials and Methods

The study cohort comprised three tissue microarrays (TMAs) containing two (in single cases three) cores of formalin-fixed and paraffin-embedded (FFPE) tumor tissue of 41 samples of PDAC and 41 samples of CC from the Institute of Pathology, Regensburg, Germany. The patient samples were randomly assigned to the TMA blocks, meaning that the TMA blocks were mixed in terms of their tumor entity to control for technical inter-measurement bias. Information regarding patient characteristics (age, sex) and tumor parameters (T stage, nodal involvement, tumor grading, resection margins) was also provided by the Institute of Pathology, Regensburg, Germany. The study was approved by the institutional ethics review board (Ethics Committee of the Technical University of Munich Faculty of Medicine, Protocol Number 403/17S).

In preparation for the measurement, indium tin oxide (ITO) conductive glass slides (Bruker Daltonik, Bremen, Germany) were coated with poly-ʟ-lysine. Three micrometer-thick sections of each TMA block were mounted onto the ITO slides. 

The sample preparation for MALDI-MSI data acquisition was performed according to a standard protocol as previously described in detail [17]. Briefly, paraffin was removed with graded xylene and ethanol washes followed by antigen retrieval using a decloaking chamber (BioCare Medical, Pacheco, CA, USA) at 110 °C for 20 min with de-ionized water as buffer medium. After air drying, the sections were subjected to on-tissue tryptic digestion. The enzyme was homogenously applied onto the tissue at a concentration of 20 µg/mL trypsin (Sequencing Grade Modified Trypsin, Promega, Madison, WI, USA) in an array-based pattern over a series of 16 passes (5 × 10^−3^ µg/mm^2^) by a pneumatic sprayer (TM Sprayer, HTX Technologies, LLC, Chapel Hill, NC, USA). Digestion was achieved by placing the slides in a humidified digestion chamber (provided by a saturated K_2_SO_4_ solution) for two hours at 50 °C. Following digestion, a α-cyano-4-hydroxycinnamic acid (HCCA) matrix (Merck KGaA, Darmstadt, Germany) was sprayed onto the tissue sections. The matrix solution consisted of 9.9 mg/mL HCCA in 70/30/0.1 (*v/v/v*) acetonitrile/de-ionized water/trifluoroacetic acid (Carl Roth GmbH, Karlsruhe, Germany). Spraying parameters were set to a linear pattern and four iterations for a final concentration of 2 × 10^−3^ µg/mm^2^.

Samples were analyzed using a rapifleX MALDI Tissuetyper time-of-flight mass spectrometer (Bruker Daltonik). The mass spectrometer was operated with positive polarity in reflector mode and spectra were acquired in the mass range of *m/z* 600–3200. Mass spectra were collected at a spatial resolution of 50 µm. Calibration was achieved by using an on-slide calibration standard (Peptide Calibration Standard II, Bruker Daltonik). 

**Figure 1:**
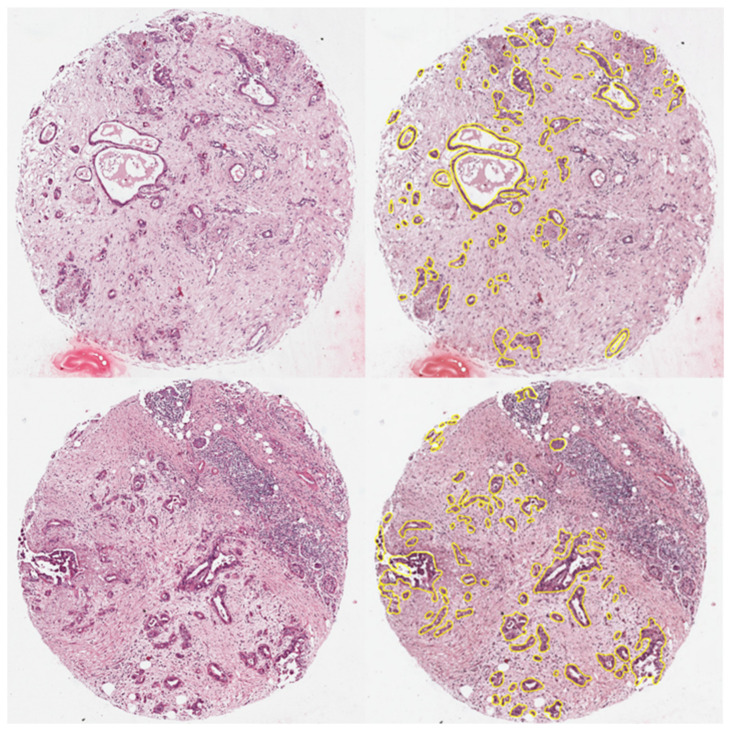
One exemplary core of a cholangiocarcinoma (CC) (top) and a pancreatic ductal adenocarcinoma (PDAC) (bottom), without (left side) and with annotation (right side).

Following data acquisition, matrix coating was rinsed off the tissue sections by means of two subsequent methanol washes. Afterward, tissue sections were stained following standard protocols for H&E staining and scanned at a magnification of 40× using a digital microscope slide scanner (Aperio CS2, Leica Biosystems, Wetzlar, Germany). These images were uploaded to the SCiLS Cloud (Bruker Daltonik, discontinued service) and meticulously annotated by a pathologist (C.B.) to mark the epithelial tumor regions of each core (Figure 1). This information was stored as SEF files, imported into SCiLS Lab software (Bruker Daltonik), and coalesced with the measurement regions to obtain spatially resolved spectral data of the annotated regions. The spectra were preprocessed according to a novel cross-normalization algorithm proposed by Boskamp et al. that includes most notably mass-dependent intensity profile normalization, peptide mass resampling, and total ion count normalization [18]. Figure S1 displays the average spectra of the PDAC and CC tumor regions. A list of 175 peaks with the highest intensities was automatically generated in SCiLS lab via the tool “Find peaks”. Additionally, information regarding tumor label (PDAC and CC, respectively), as well as case ID, were compiled in an attribute table. Spectral intensity for each peak and metadata were then exported and saved as *.csv files for further analysis with the open-source programming language python (version 3.8). 

Tentative identification of the *m/z* features was carried out by MS/MS measurement on whole-mount PDAC tissue sections. The measurements were performed in situ using a Rapiflex in LIFT mode and a timsTOFflex (for *m/z* = 850.4) mass spectrometer (Bruker Daltonik). The fragmentation spectra of selected *m/z* values were collected in positive ionization mode, using a smartbeam M5 flat laser configuration at a frequency of 200 Hz, and a beam scan of 25 µm^2^. Laser power boost was set at 80%. The fragmentation spectra of *m/z* = 850.4 were also generated in positive ionization mode. Laser power for fragmentation was set at 70%, for a laser frequency of 5000 Hz, and a beam scan of 25 µm^2^. For the identification, the MS/MS spectra (Figures S3–S8) were submitted to the MASCOT MS/MS Ion Search, where the SwissProt database was searched to match tryptic peptide sequences to the respective intact proteins, defining *homo sapiens* as the taxonomic class. The MS/MS spectrum search parameters included a mass tolerance of 1 Da, MS/MS tolerance of ±1 Da, up to two missed cleavages, methionine oxidation, protein *N*-terminal acetylation, and proline oxidation as variable modifications. 

The statistical analysis of patient characteristics and tumor parameters (Table 1) was performed in Microsoft Excel (2016, Microsoft Corporation, Redmond, WA, USA). After the normal distribution of the continuous variable had been verified by the Kolmogorov–Smirnov test, the Student’s *t*-test was applied to look for a significant difference between the mean values of both tumor entities. For the categorical variables, the Chi-squared test was used to detect significant differences in distribution between both tumor types. The significance level for all tests was set at *p* = 0.05. 

## 3. Results

### 3.1. Patient Characteristics

Table 1 summarizes patient features of the two tumor entities. Significant differences can be identified in terms of gender (73.2% male patients for pancreatic carcinoma vs. 51.2% of male patients for cholangiocarcinoma, *p* = 0.04), tumor stage (26.8% pT1/pT2 for pancreatic carcinoma vs. 70.7% pT1/pT2 for cholangiocarcinoma and correspondingly 73.2% pT3/pT4 for pancreatic carcinoma vs. 29.3% pT3/pT4 for cholangiocarcinoma, *p* < 0.01), as well as for nodal stage (95.1% pN+ for pancreatic carcinoma vs. 63.4% pN+ for cholangiocarcinoma and correspondingly 4.9% pN− for pancreatic carcinoma vs. 36.6% pN− for cholangiocarcinoma, *p* < 0.01). For the variables age, tumor grading, and resection status no significant differences could be found.

### 3.2. Supervised Classification 

Based on the metadata, supervised analysis was carried out using three different machine learning algorithms, that is support vector machine (SVM), k-nearest neighbors (KNN), and gradient boosting (GB). For non-linear SVM (kernel = rbf) and KNN, 80% of spectra (47.364 individual spectra in total) were used for hyperparameter tuning (hyperparameters C (regularization parameter) = 505 and gamma (kernel width) = 0.055 for SVM; k (number of neighbors) = 2 for KNN) to optimize model performance prior to the classification task itself. The remaining data (20% of the spectra for SVM and KNN; 100% of spectra for GB as no prior hyperparameter tuning was needed) were subsequently randomly split into a training set and a test set at a ratio of 70 to 30 to ensure independent and reliable evaluation of model performance on unknown data. To reduce the potential impact of overfitting, a tree-based feature selection step that was the same for all algorithms was integrated into all three classification pipelines at the training/test set level. On average, the 50–60 most important features (that is *m/z* values) were selected and used for classification. 

**Figure 2:**
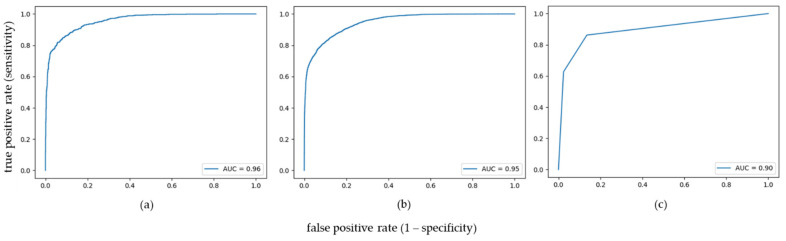
ROC curves for the binary classification task of differentiating between pancreatic ductal adenocarcinoma and cholangiocarcinoma using (**a**) support vector machine, (**b**) gradient boosting, and (**c**) k-nearest neighbors.

The optimized and trained algorithms were applied to the test set on a pixel level. Additional assessment of model performance on subject level was calculated as follows: if more than 50% of spectra of all cores of a case were classified correctly, the case was defined as correctly classified. SVM and KNN yielded an area under the curve (AUC) of 0.96 and 0.90 on pixel level as well as correctly classified 65 and 68 out of 76 cases (85.53 and 89.47%), respectively. GB reached an AUC of 0.95 on pixel level and correctly classified 66 out of 79 cases (83.54%) (Figure 2).

Additional calculation of the performance metrics on a pixel level showed that SVM reached the highest accuracy of 0.91 and was closely followed by GB and KNN with accuracies of 0.88 and 0.86, respectively (Table 2). A closer look at the other metrics revealed that SVM and GB performed better for the classification of CC. KNN performed equally well for both tumor types.

This observation could possibly be due to the fact that the prevalence of tumor spots was shifted in favor of cholangiocarcinoma with a ratio of about 70/30. To correct this disparity, one in three cases of cholangiocarcinoma was removed from the cohort, resulting in a ratio of CC:PDAC of 54/46 for the new reduced dataset. Repeated calculations on this reduced dataset yielded similar good results with an AUC of 0.95 and 96.23% correctly classified cases (51/53) for GB, an AUC of 0.94 and 94.12% correctly classified cases (48/51) for SVM and an AUC of 0.90 and 92.16% correctly classified cases (47/51) for KNN. Table 3 summarizes the performance metrics on pixel level for the reduced dataset. The results for accuracy again remained very similar, whereas this time all algorithms now conversely had more difficulty in correctly classifying the tumor spots of cholangiocarcinoma, especially KNN. In contrast to KNN, this difference is not so pronounced for SVM and GB (Table 3). 

### 3.3. Discriminative m/z Values

Given Python’s built-in code to retrieve feature importance for tree-based machine learning algorithms, a list of the most significant features for the gradient-boosting classifier was created for the whole as well as the reduced dataset. The ranking of features is based on the mean decrease in impurity (or Gini importance) that indicates the extent to which the variable/feature adds to the homogeneity/purity of the tree’s nodes and leaves. The ten most important features for calculation on the whole and the balanced dataset were as follows:

- *m/z* 3086.53, 3085.52, 850.43, 3101.51, 2104.07, 2073.05, 3102.53, 2056.07, 1199.67, 3100.51, for calculation on the whole dataset
- *m/z* 2692.33, 3086.53, 1138.55, 1199.67, 1460.70, 816.38, 3085.52, 850.43, 2073.05, 1581.75 for calculation on the reduced dataset

**Figure 3:**
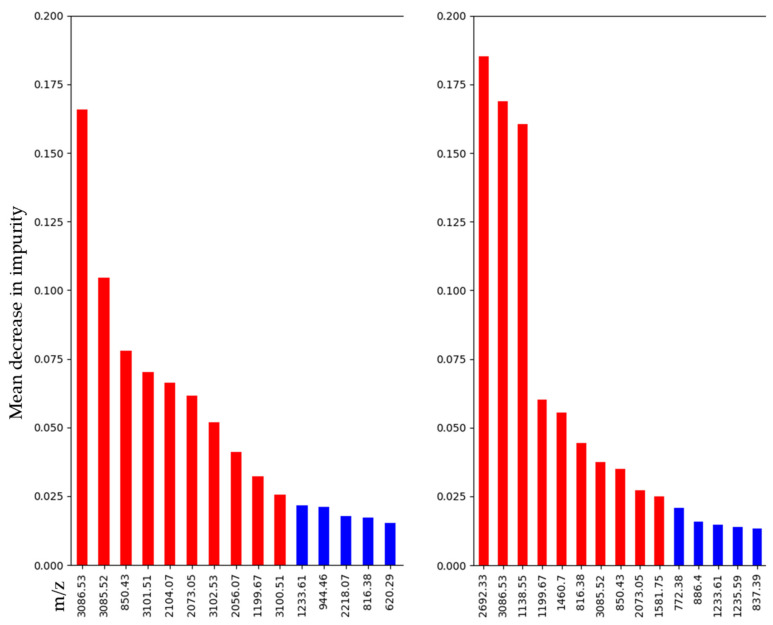
Feature importance using the mean decrease in the impurity of gradient-boosting classification on whole dataset (left side) and balanced dataset (right side) (top 10 features with red bars, others with blue bars).

There is some variability in the selection and ranking of variables, but most variables, nevertheless, appear in both lists. The lists of the top 15 important features are presented in Figure 3. All the features used for gradient-boosting classification are displayed in Supplementary Figures S2 and S3. Comparative ion images from PDAC and CC for the four most discriminative *m/z* values are integrated in Figure S4.

### 3.4. Tentative m/z Identification

Some of the *m/z* features with a higher impact on gradient-boosting classification performance could successfully be isolated and fragmented during the MS/MS measurement. *m/z* 850.4 and *m/z* 944.5 gave a match for histone H2A, *m/z* 1105.5, *m/z* 2056.0, and *m/z* 2073.0 for the collagen alpha-1(I) chain, and *m/z* 1198.7 for actin/cytoplasmic 1 (Table 4). 

## 4. Discussion

Cholangiocarcinoma (CC) and pancreatic ductal adenocarcinoma (PDAC) are both malignant epithelial neoplasms with glandular/ductal differentiation and profound stromal desmoplasia. To date, it is essentially impossible for pathologists to draw a clear distinction between adenocarcinomas of biliary or pancreatic origin based on histology alone due to their close morphological resemblance. This well-known diagnostic dilemma can be typically encountered when dealing with a mass lesion located in the pancreatic head which is the most frequent site of manifestation of pancreatic carcinomas accounting for 75% of all pancreatic carcinomas [19]. There are a few tumor features that can provide valuable hints about tumor origin. This includes the presence of an in situ component, such as biliary intraepithelial neoplasia (BilIN) or pancreatic intraepithelial neoplasia (PanIN), as well as the evaluation of the tumor epicenter in relation to anatomical structures. In practical terms, this means that tumors associated with BilIN and a circumferential and symmetric involvement of the distal common bile duct are most likely to be cholangiocarcinomas, whereas tumors associated with PanIN and a tumor focus away from the distal common bile duct favor a pancreatic carcinoma [20,21]. An unambiguous identification of tumor derivation, however, cannot be deduced therefrom. To make a definitive diagnosis, additional clinical information, in particular regarding diagnostic imaging results, are needed. 

MALDI-MSI presents a convenient analytical tool to address this issue by enabling the detection and visualization of biomolecules in intact tissue sections across different tissue/tumor types. We used tryptic peptide signatures confined to tumor regions to create machine learning models aimed at differentiating between CC and PDAC. All classifiers achieved very good results on a pixel level, as well as on a case level. On a pixel level, the support vector machine (SVM) was leading with an AUC of 0.96 and an accuracy of 0.91, closely followed by gradient boosting (GB) with an AUC of 0.95 and an accuracy of 0.88 and a little behind KNN with an AUC of 0.90 and an accuracy of 0.86. Additionally, more than 80% of cases were classified correctly (89.47, 85.53, and 83.54% for KNN, SVM, and GB, respectively). However, the prevalence of tumor spots was skewed towards cholangiocarcinoma with a ratio of about 70/30. We, therefore, decided to repeat all calculations after dropping two-thirds of CC cases to obtain a balanced dataset in terms of data points. The results of the calculations on this reduced dataset were also very good, this time led by GB (AUC 0.95, accuracy 0.88, and 96.23% correctly classified cases) and followed by SVM (AUC 0.94, accuracy 0.89, and 94.12% correctly classified cases) and KNN (AUC 0.90, accuracy 0.85, and 92.16% correctly classified cases). This finding is of particular importance as it confirms that the performance of the classification models is indeed trustworthy and not only based on random guessing but is robust enough to accommodate imbalances in the different classes in the dataset, without compromising the accuracy of the classification. However, it turns out that the classification of CC on the balanced dataset seems to be more difficult than the classification of PDAC. On the non-reduced data set it was rather the opposite, but it must be considered that data for the training step were strongly predominated by CC. It is therefore more challenging for a model to learn the characteristics of the minority class, and to differentiate samples from this class from the majority class. The challenge of correctly categorizing CC might be at least partially due to the heterogeneity of this tumor type. There are different anatomical subtypes (intrahepatic, perihilar, and distal) that are distinguished by distinct genetic alterations, divergent clinical presentations, and varying therapeutic options [22]. In this retrospective pilot study, no distinction was made between these tumor subtypes. We acknowledge that in future projects it is essential to build TMAs containing sufficiently enough and about the same number of samples of each category to verify this assumption. Alternatively, it could be advisable to form a provisional exclusion category (neither PDAC nor CC) which can then be scrutinized and subclassified in more detail in future analyses.

The good results generated by the machine learning algorithms on mass spectrometry data highlight that MALDI-MSI is equal or even superior to established procedures. On top of that, only a single tissue section that stays intact throughout the analysis is required and, thus, can afterwards be used for staining (H&E or IHC). All classifiers performed similarly well on pixel analysis, as well as on a case level, as previously discussed. The function of the tree-based algorithms for extracting the most important features for building the model ranks the features by their weight in the decision-making of the classification model. The list of features’ importance differs to some extent between classification on the whole unbalanced and the reduced balanced dataset; still, a lot of features are shared between them, some just being isotopes of the same peptide (supplementary Figures S1 and S2). Part of the difference is therefore due to the different weighting of the features. The fact that some of these *m/z* values (*m/z* 3085.52 and 3086.53 as well as 3100.51, 3101.51, and 3102.53) are isotopes indicates that they may indeed have biological relevance to divergent tumorigenesis in PDAC and CC. Among the *m/z* features with a higher relevance in building up the GB classifier, some could successfully be isolated and fragmented during the MS/MS experiment. As summarized in Table 4, *m/z* 850.4 and *m/z* 944.5 correspond to histone H2A, *m/z* 1105.5, *m/z* 2056.0, and *m/z* 2073.0 correspond to collagen alpha-1(I) chain, and *m/z* 1198.7 corresponds to actin. For these proteins, there are already multiple studies in connection with PDAC to be found in the literature. H2A.Z isoforms were reported to be overexpressed in PDAC cell lines and PDAC tissues, resulting in tumor growth and resistance to chemotherapy [23]. In this study, Avila-Lopez et al. demonstrated that the depletion of H2A.Z isoforms promotes tumor senescence, increases sensitivity to the chemotherapeutic drug gemcitabine, and decreases tumor growth in a mouse xenograft model in vivo. Similarly, proteins involved in the modification of the tumor microenvironment, particularly in the extracellular matrix, play a crucial role in PDAC progression. Tian and coworkers conducted a thorough analysis of pancreatic extracellular matrix composition comprising murine and human samples of normal pancreatic tissue, as well as different pathological conditions including chronic pancreatitis, pancreatic intraepithelial neoplasia (PanIN), and PDAC [24]. Their results illustrate the increasing abundance and complexity of extracellular matrix proteins, among them fibrillar collagens such as COL1A1, 1A2, and 3A1, during tumor progression. Moreover, they highlighted the importance of distinguishing between stromal cell-derived and cancer-cell-derived matrix proteins as they correlate with different survival rates. In a follow-up study, they were able to reveal the tumor-suppressive effect of cancer-cell-derived fibrillar collagen COL1A1 which, however, requires prior cleavage of the C-terminal pro-domain by the procollagen C-proteinase bone morphogenetic protein1 (BMP1) [25]. The detection of the partially uncleaved C-terminal pro-domains of fibrillar collagens in the extracellular matrix of PDAC therefore seems to be attributed to reduced pro-collagen C-proteinase activity. Further studies are definitely needed to unravel the complex interactions in extracellular matrix formation, nonetheless, it is clear that collagens are essential components in the pathogenesis of PDAC. Besides different types of collagens, variants of the structural protein actin were shown to be differentially expressed in pancreatic carcinomas with or without lymphatic vessel invasion (actin/cytoplasmic 1 and collagen alpha-2(I) chain), as well as pancreatic carcinomas, with and without blood vessel invasion (collagen alpha-2(I) chain) [26]. All these reports underline the assumption that the discriminative features found here indeed hold a biological significance. Future efforts on our part will focus, among other things, on identifying further *m/z* features. 

Many other biomarker studies have already been carried out to address the diagnostic dilemma of distinguishing between CC and PDAC. On the one hand, part of the analyses was confined to the evaluation of biomarkers already established in today’s routine diagnostics. On the other hand, some scientific studies aimed at the discovery of novel biomarkers, mainly applying mass spectrometry methods. The reported panels of immunohistochemical markers achieved good to modest results in terms of their diagnostic performance in distinguishing between CC and PDAC. Kälsch et al., for instance, were able to correctly classify 85% of intrahepatic cholangiocarcinoma and 75% of metastatic pancreatic ductal adenocarcinoma by means of annexin-10 in univariate analysis [27,28]. They, however, indicated that these results were gained using a semi-quantitative scoring system analogous to IRS (immunoreactivity score) with a cut-off value of one. This threshold implies that only a weak staining intensity of more than 5% of tumor cells is sufficient enough for a diagnosis of PDAC. In daily practice, this can be delusive given the not uncommon observation of staining artifacts, especially when dealing with poorly fixated or otherwise altered tissue specimens. This drawback of the per se promising biomarker candidate is attenuated by combining it with other markers assigned with higher cut-off values. The combination of annexin-10, annexin-1, and cytokeratin 17 finally yielded an accuracy of 84%. Its widespread application in routine diagnostics, however, suffers from the requirement of performing a semi-quantitative analysis for each of the proposed biomarkers, which can be quite time-consuming and inherits the risk of significant intra- and interobserver discrepancies. 

Lok et al. proposed a combination of four immunohistochemical markers to differentiate between intrahepatic CC and PDAC [29]. According to their results, a staining pattern of S100P−/pVHL+/MUC5AC−/CK17− is found in intrahepatic CC whereas the configuration of S100P+/pVHL−/MUC5AC+ or −/CK17+ portends a PDAC. These panels reached excellent specificity values of 100% (cholangiocarcinoma panel) and 97.5% (pancreatic cancer panels). In contrast, the sensitivity merely achieved moderate to subdued values of 41.4 (CC panel), 36.7, and 18.3% (PDAC panel). Therefore, this marker panel can be at best supportive but by no means sufficient to make a reliable diagnosis. 

Another biomarker combination, namely N-cadherin and HPC2 (human pancreatic cancer fusion #2), was subjected to IHC examination by Hooper et al. [30]. With the same objective to distinguish metastatic PDAC from CC, the marker constellations HPC2-/N-cadherin+ and HPC2+/N-cadherin- were regarded to be indicative of cholangiocarcinoma and pancreatic carcinoma, respectively. Using this marker set, Hooper et al. achieved a sensitivity of 45% and a specificity of 93% for cholangiocarcinoma, and a sensitivity of 60% and a specificity of 80% for metastatic pancreatic carcinoma. Similar to the results gained by Lok et al., the biomarker pair somewhat failed to provide sufficiently high values for sensitivity while obtaining good values for specificity. 

The same applies to a study performed by Hrudka et al., in which immunohistochemical staining for the transcription factor FOXF1 proved to be quite specific (87%), but only in the distinction of peripheral intrahepatic cholangiocarcinoma versus hilar/extrahepatic cholangiocarcinoma and metastatic PDAC [31]. An investigation to distinguish the latter two entities was not conducted. Moreover, the low sensitivity of 65% they reported is inadequate for use in routine diagnostics. 

In contrast, Somorácz et al. could obtain a sensitivity of 94/81% and a specificity of 100/89% depending on whether a quantitative or qualitative approach was chosen for the IHC interpretation of the marker agrin [32]. These figures seem quite convincing, yet there are some methodological drawbacks complicating its introduction into routine diagnostics. On the one hand, quantitative evaluation was carried out by digital morphometry which requires the additional scanning of the slides as well as the availability of the software tool. On the other hand, qualitative evaluation was based on the criterion as to whether the largest continuous agrin-negative area was smaller or greater than the 10× visual field. This, however, can become problematic as poor fixation and consequently areas of false-negative staining are a well-known obstacle in daily practice, especially in the center of large tumors. Also, an area sufficiently large to make that distinction is hardly ever seen in a pre-therapeutic biopsy, which is in a setting where differentiating these two tumor types is the most crucial regarding treatment decision. As far as the results themselves are concerned, it is worth mentioning that a subdivision of the diagnostic performance according to the degree of differentiation was not provided, although the level of differentiation was shown to have a major impact on agrin expression. Thus, it remains uncertain whether loss or preservation of agrin expression is as clear-cut for the distinction between well-differentiated cholangiocarcinomas and metastatic pancreatic carcinomas as it is for poorly differentiated ones. 

A general disadvantage of most immunohistochemical panels is their need for several different stains that are usually performed utilizing one tissue section per stain. However, the scarcity of tumor tissue in small pre-therapeutic biopsies that are meant to confirm a tentative diagnosis is often the limiting factor. An alternative approach to solve this dilemma is the use of molecular analyses. To our knowledge, however, the only papers explicitly addressing the discrimination of pancreatic cancer and cholangiocarcinoma based on molecular markers are the ones published by Fasanella et al. [33] and Mosbeh et al. [34]. In the first study, K-Ras-2 exon 1 PCR-amplified DNA of 33 cytological samples of pancreatic cancer and extrahepatic cholangiocarcinoma was sequenced to search for differences in mutation rate. A K-Ras mutation was found in 87.5 and 5.9% of pancreatic carcinoma and extrahepatic cholangiocarcinoma, respectively. Regarding pancreatic carcinoma, the resulting sensitivity and specificity were 87.5 and 94.0%, respectively. Still, these values do not reveal the whole truth as no cases of intrahepatic cholangiocarcinoma were included. This is crucial though, because of the major differences in K-Ras mutation rates between intra- and extrahepatic cholangiocarcinomas, and even between the small-duct and large-duct types within the group of intrahepatic cholangiocarcinomas [35,36]. In the second study by Mosbeh et al., a joint approach consisting of molecular and immunohistochemical analysis for the tumor suppressor gene BAP1 was performed. Indeed, the frequency of BAP1 loss was significantly higher in intrahepatic cholangiocarcinoma when compared to extrahepatic cholangiocarcinoma and pancreatic carcinoma. However, the loss of BAP1 was only reported in 50% of cases of intrahepatic cholangiocarcinoma, which still leaves the question of how to classify the other 50% of cases, especially when considering that a small portion of pancreatic carcinomas also shows this loss. 

In the current study, we used MALDI-MSI which offers many benefits in terms of saving scarce tissue material and overcoming intra- and interobserver disagreement using classification algorithms. Moreover, MALDI-MSI is an untargeted approach that does not require prior knowledge of possible analytes; hence, this technique is unbiased. As the TMA blocks were mixed in terms of tumor entity, we could control technical bias based on batch effects. The additionally implemented tree-based feature selection step helped to reduce the risk of overfitting, which makes the classifier robust for application on other unseen datasets. It remains to be clarified in future studies whether MALDI-MSI can also achieve reliable and reproducible results with tumor tissue of biopsies and metastases as well as from different collection sites. 

## 5. Conclusions

The results of this pilot study show that MALDI-MSI is a suitable tool for the reliable classification of histomorphologically similar tumor types. In particular, the findings confirm the overall eligibility of this technique for the comprehensive and in-depth analysis of tumors of the pancreas and the biliary tree, where an accuracy of about 90% could be achieved. Focusing on the molecular features that play a key role in the differentiation of both entities could pave the way for unraveling the complex mechanisms and interactions of pancreatobiliary tumor biology and promote the development of novel diagnostic tools and future therapeutic targets. 

## Figures and Tables

**Table 1:** Characteristics of the study population.

	Pancreatic Carcinoma (PDAC) (*n* = 41)	Cholangiocarcinoma (CC) (*n* = 41)	*p* Value
**Age in years** **(mean ± SD)**	66 ± 11.4	64 ± 11.9	0.49 †
**Male sex (%)**	73.2	51.2	0.04 *‡
**T stage**			
**pT1/pT2 (%)**	26.8	70.7	< 0.01 *‡
**pT3/pT4 (%)**	73.2	29.3	
**Nodal involvement**			
**pN+ (%)**	95.1	63.4	< 0.01 *‡
**pN− (%)**	4.9	36.6	
**Tumor grading**			
**G1/G2 (%)**	51.2	51.2	1.00 ‡
**G3 (%)**	48.8	48.8	
**Resection margins**			
**R0 (%)**	53.7	73.2	0.07 ‡
**R1 (%)**	46.3	26.8	

* Statistically significant; † student’s *t*-test; ‡ Chi-squared test.

**Table 2:** Performance metrics for the binary classification task of differentiating between pancreatic ductal adenocarcinoma and cholangiocarcinoma using gradient boosting, support vector machine, and k-nearest neighbors on the whole dataset.

Classification Algorithm	Accuracy		FNR *		FPR **		TNR ***		TPR ****	
Class	CC	PDAC	CC	PDAC	CC	PDAC	CC	PDAC	CC	PDAC
Support vector machine	0.91	0.91	0.03	0.24	0.24	0.03	0.76	0.97	0.97	0.76
Gradient boosting	0.88	0.88	0.02	0.34	0.34	0.02	0.67	0.98	0.98	0.67
K-nearest neighbors	0.86	0.86	0.14	0.14	0.14	0.14	0.86	0.87	0.87	0.86

* False negative rate; ** False positive rate; *** True negative rate; **** True positive rate.

**Table 3:** Performance metrics for the binary classification task of differentiating between pancreatic ductal adenocarcinoma and cholangiocarcinoma using gradient boosting, support vector machine, and k-nearest neighbors on the balanced dataset.

Classification Algorithm	Accuracy		FNR *		FPR **		TNR ***		TPR ****	
Class	CC	PDAC	CC	PDAC	CC	PDAC	CC	PDAC	CC	PDAC
Support vector machine	0.89	0.89	0.16	0.07	0.07	0.16	0.93	0.84	0.84	0.93
Gradient boosting	0.88	0.88	0.19	0.07	0.07	0.19	0.93	0.82	0.82	0.93
K-nearest neighbors	0.85	0.85	0.26	0.07	0.07	0.26	0.93	0.74	0.74	0.93

* False negative rate; ** False positive rate; *** True negative rate; **** True positive rate.

**Table 4:** Tentative MS/MS identification.

Observed *m/z*	Mr (Expect)	Mr (Calc)	Error (Da)	Peptide Sequence	Protein (UniProtKB Protein Accession Number)	Modifications
850.4	850.5	849.5	0.03	R.HLQLAIR.N	Histone H2A (B2R5B3)	
944.5	944.6	943.5	0.10	R.AGLQFPVGR.I	Histone H2A (B2R5B3)	
1105.5	1105.5	1104.6	0.17	R.GVQGPPGPAGPR.G	Collagen alpha-1(I) chain (P02452)	Oxidation (*p*)
2056.0	2056.0	2056.0	0.43	K.TGPPGPAGQDGRPGPPGPPGAR.G	Collagen alpha-1(I) chain (P02452)	Oxidation (*p*)
2073.0	2073.0	2072.0	0.24	K.GSPGADGPAGAPGTPGPQGIAGQR.G	Collagen alpha-1(I) chain(P02452)	
1198.7	1198.7	1197.7	0.22	R. AVFPSIVGRPR.H	Actin *	

* possible underlying isoforms: ACTA1 (P68133) or ACTA2 (P62736), ACTAB (P60709), ACTG1 (P63261), ACTG2 (P63267), POTEI (P0CG38), POTEKP (Q9BYX7), POTEF (A5A3E0) or POTEE (Q6S8J3).

## Data Availability

The data (including python code and mass spectrometry raw data) presented in this study are available on request from the corresponding author.

## Supplementary Materials

The following supporting information can be downloaded at: https://www.mdpi.com/article/10.3390/molecules27113464/s1, Figure S1: Sum spectra of annotated tumor regions of PDAC and CC, Figure S2: Feature importance using mean decrease in impurity of gradient boosting classification on the whole dataset, Figure S3: Feature importance using mean decrease in impurity of gradient boosting classification on the reduced dataset, Figure S4: Ion images of top four features with highest discriminative power in gradient boosting classification on representative PDAC and CC cores, Figure S5: MS/MS fragmentation spectra of *m/z* 850.4MS/MS fragmentation spectra of *m/z* 850.4 using timsTOF flex, Figure S6: MS/MS fragmentation spectra of *m/z* 944.5 using RapifleX, Figure S7: MS/MS fragmentation spectra of *m/z* 1105.5 using RapifleX, Figure S8: MS/MS fragmentation spectra of *m/z* 2056.0 using RapifleX, Figure S9: MS/MS fragmentation spectra of *m/z* 2073.0 using RapifleX, Figure S10: MS/MS fragmentation spectra of *m/z* 1198.7 using RapifleX.

## Sample Availability

Samples of the compounds are available from the authors by request within the scope of institutional legal regulations.
