# Supplementary material for: MALDI Mass Spectrometry Imaging for the Distinction of Adenocarcinomas of the Pancreas and Biliary Tree
Source: Molecules. 2022 May 27;27(11):3464. doi: 10.3390/molecules27113464 (PMC9182561; doi:10.3390/molecules27113464)
Supplement: Supplementary file 1 [file molecules-27-03464-s001.zip › supplementary_material_revised.pdf]

Supplementary Materials

MALDI Mass Spectrometry Imaging for the Distinction of Adenocarcinomas of the Pancreas and Biliary Tree

Christine Bollwein [1,*,†], Juliana Pereira Lopes Gonçalves [1,†], Kirsten Utpatel [2], Wilko Weichert [1] and Kristina Schwamborn [1]

[1] Institute of Pathology, School of Medicine, Technical University of Munich, Trogerstraße 18, 81675 Munich, Germany; juliana.goncalves@tum.de (J.P.L.G.); wilko.weichert@tum.de (W.W.); kchwamborn@tum.de (K.S.)
[2] Institute of Pathology, University of Regensburg, Franz-Josef-Strauß-Allee 11, 93053 Regensburg, Germany; kirsten.utpatel@klinik.uni-regensburg.de
* Correspondence: christine.bollwein@tum.de; Tel.: +49-89-4140-9336
† These authors contributed equally to this work.

Figure S1 Sum spectra of annotated tumor regions of PDAC and CC 2

Figure S2. Feature importance using mean decrease in impurity of gradient boosting classification on the whole dataset 3

Figure S3. Feature importance using mean decrease in impurity of gradient boosting classification on the reduced dataset 4

Figure S4. Ion images of top four features with highest discriminative power in gradient boosting classification on representative PDAC and CC cores 5

Figure S5. MS/MS fragmentation spectra of m/z 850.4 using timsTOF flex........ 6

Figure S6. MS/MS fragmentation spectra of m/z 944.5 using RapifleX........ 6

Figure S7. MS/MS fragmentation spectra of m/z 1105.5 using RapifleX........ 7

Figure S8. MS/MS fragmentation spectra of m/z 2056.0 using RapifleX........ 7

Figure S9. MS/MS fragmentation spectra of m/z 2073.0 using RapifleX........ 8

Figure S10. MS/MS fragmentation spectra of m/z 1198.7 using RapifleX........ 8

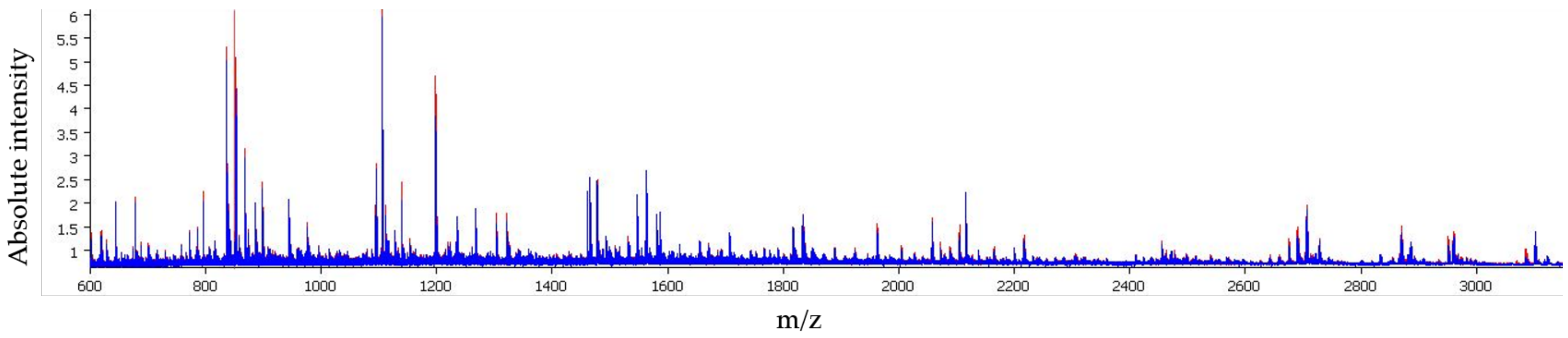

Figure S1. Sum spectra of annotated tumor regions of PDAC in red and CC in blue.

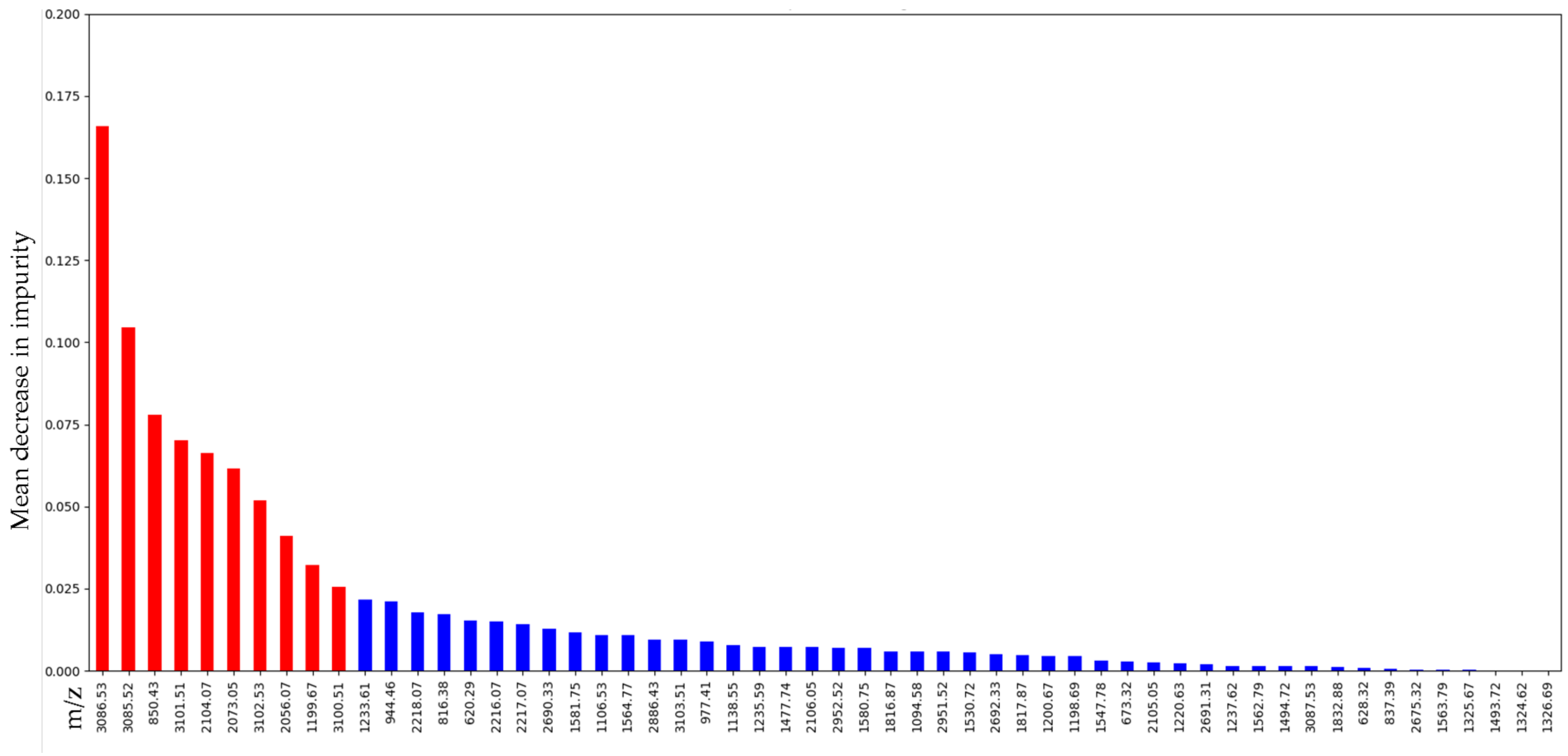

Figure S2. feature importance using mean decrease in impurity of gradient boosting classification on whole dataset (top 10 features with red bars, others with blue bars).

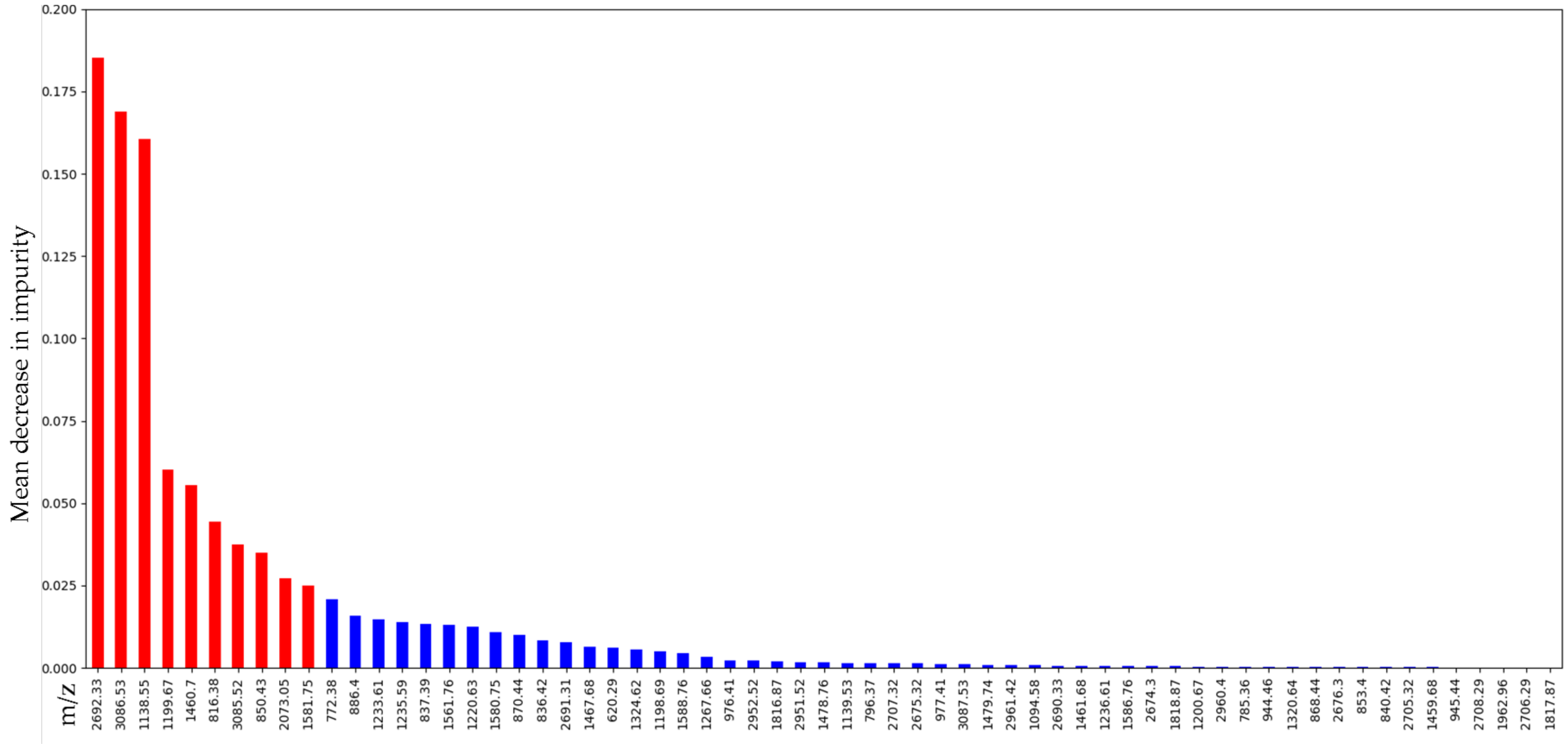

Figure S3. feature importance using mean decrease in impurity of gradient boosting classification on reduced dataset (top 10 features with red bars, others with blue bars).

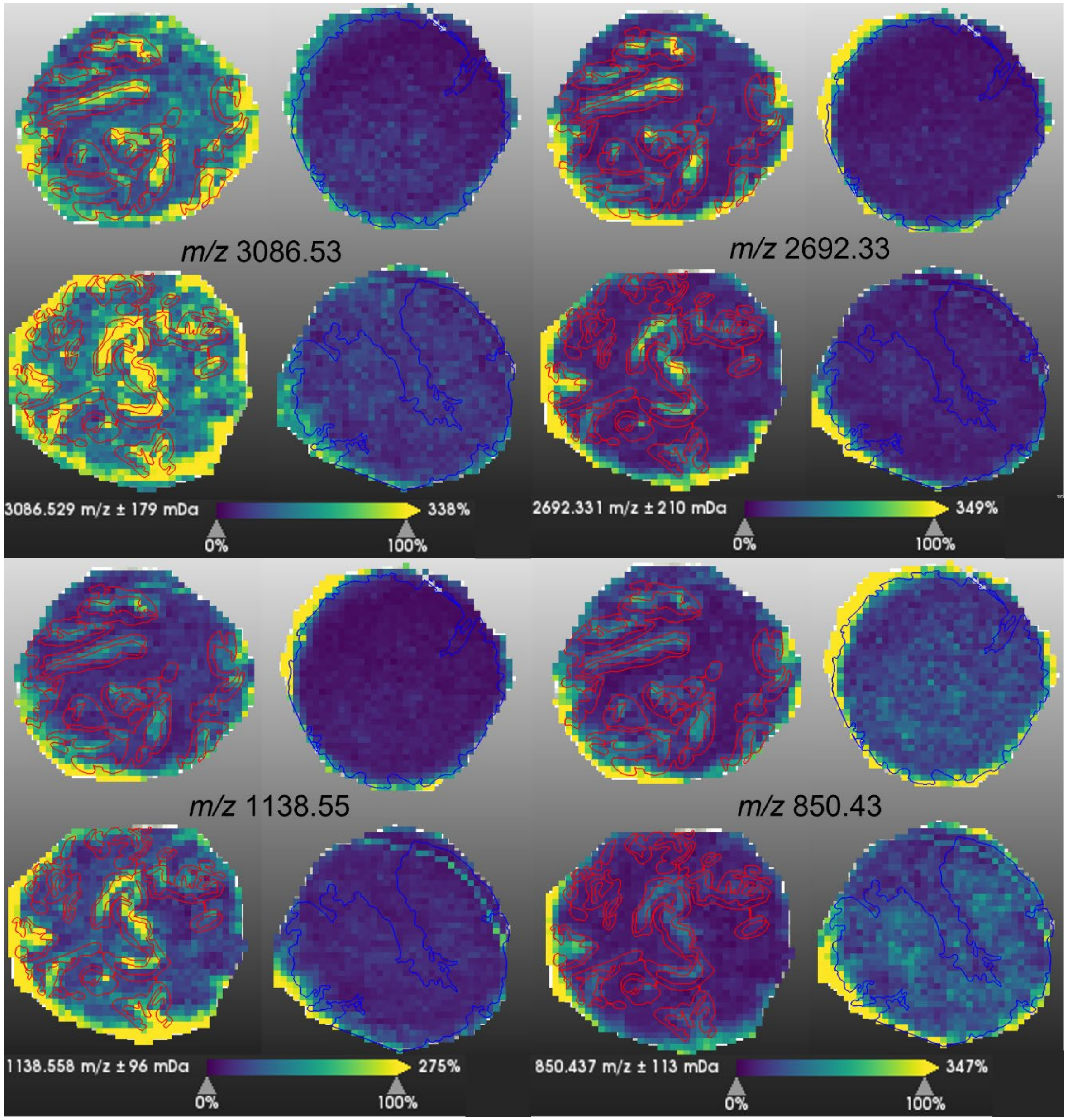

Figure S4. Ion images of m/z 3086.53 (upper left), m/z 269233 (upper right), m/z 1138.55 (lower left) and m/z 850.43 (lower right) with highest discriminative power in GB classification on representative PDAC (first and third column from left) and CC (second and forth column from left) cores.

The fragmentation spectra Figure S3-S8 were used to create the fragmentation ion list, submitted to MASCOT search for protein sequence identification.

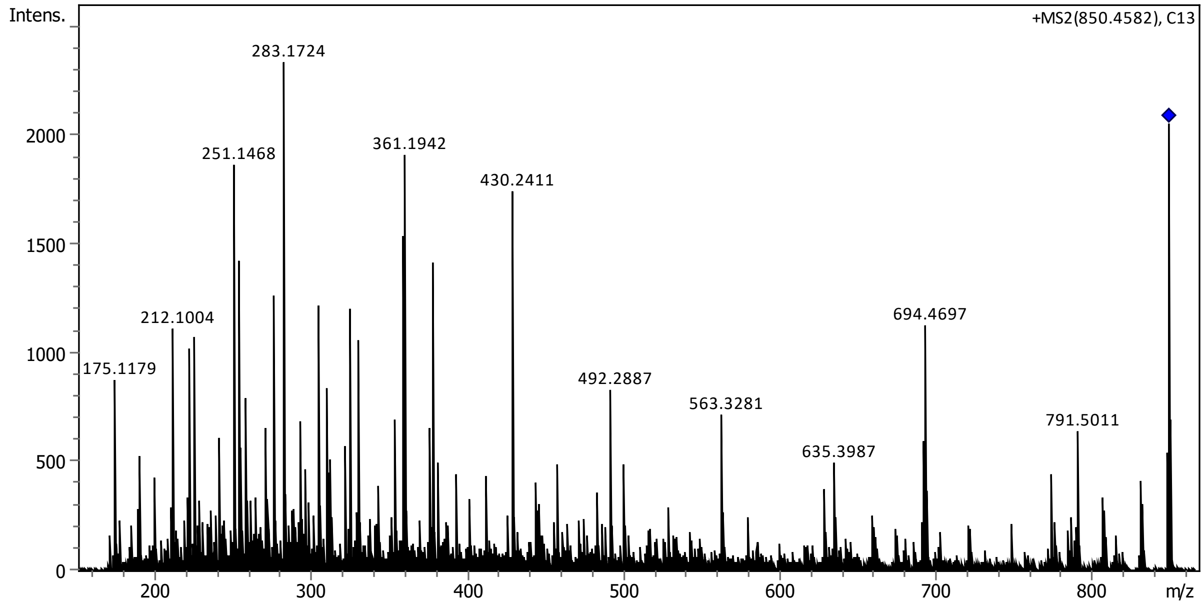

Figure S5. Fragmentation spectra of *m*/*z* = 850.4, using timsTOF flex

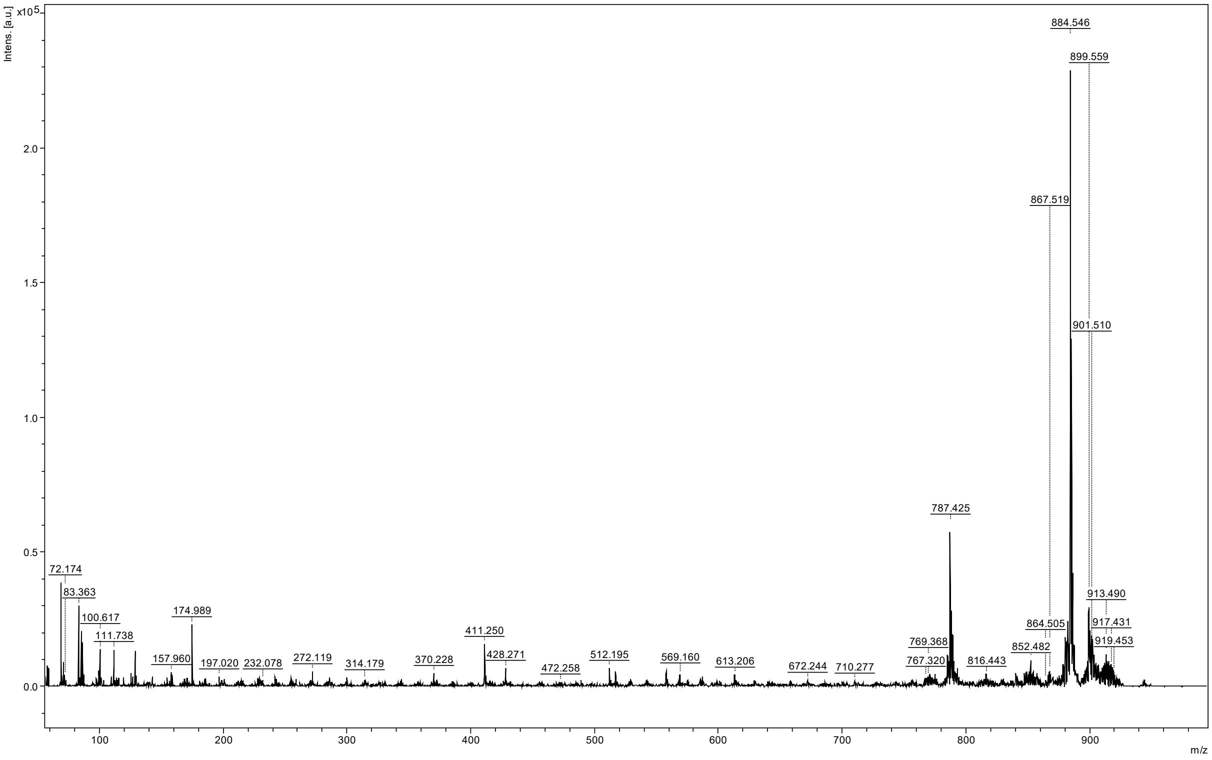

Figure S6. Fragmentation spectra of *m*/*z* = 944.5, using RapifleX

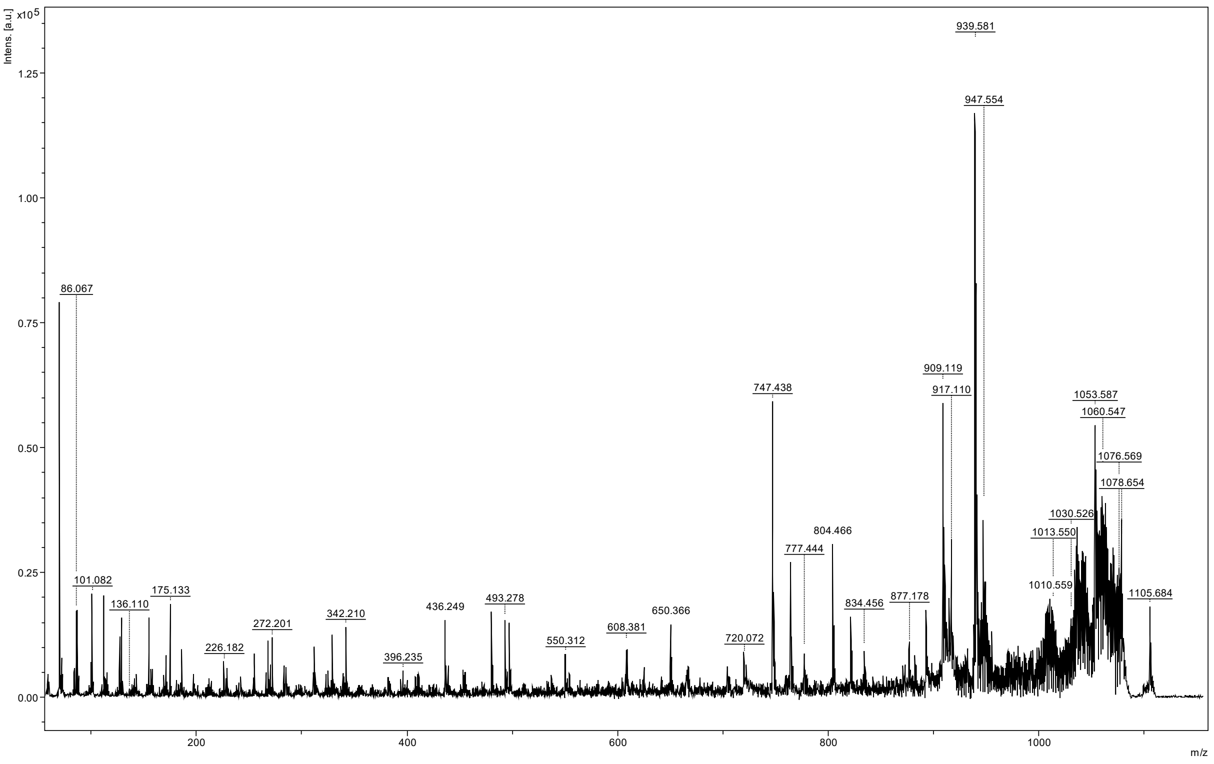

Figure S7. Fragmentation spectra of *m/z* = 1105.5 using RapifleX

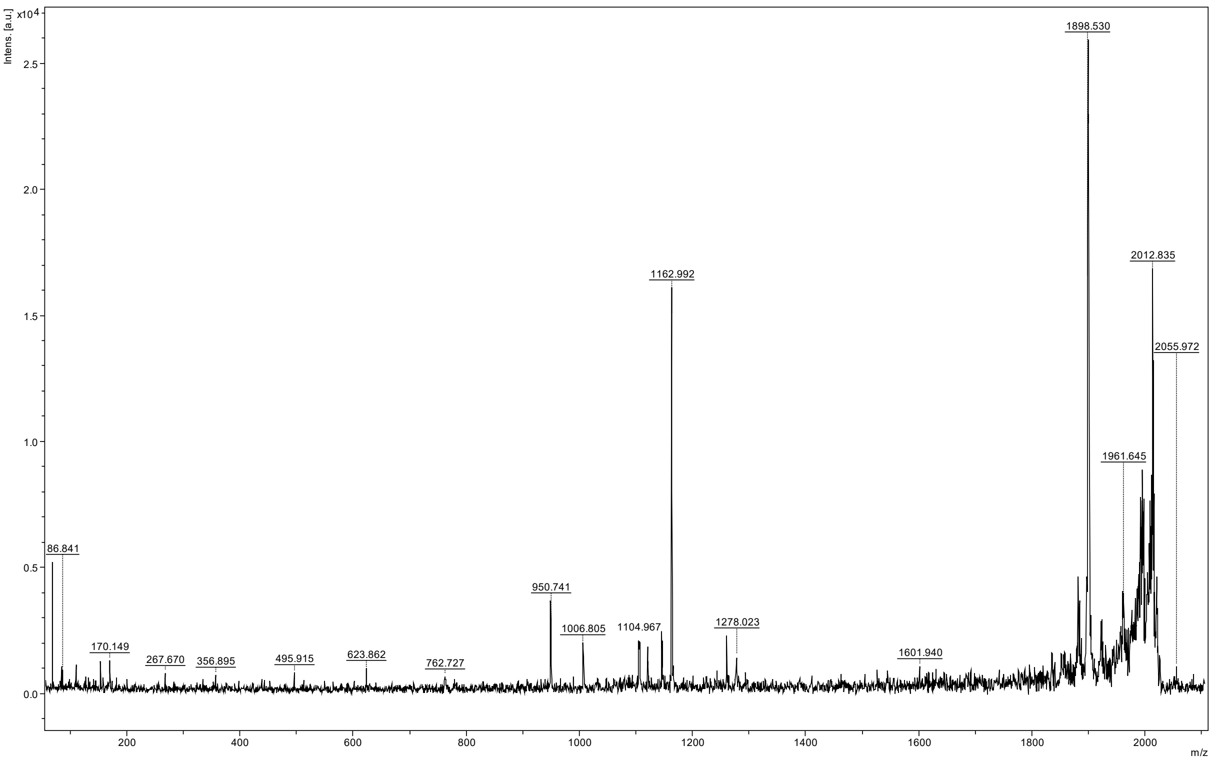

Figure S8. Fragmentation spectra of *m/z* = 2056.0 using RapifleX

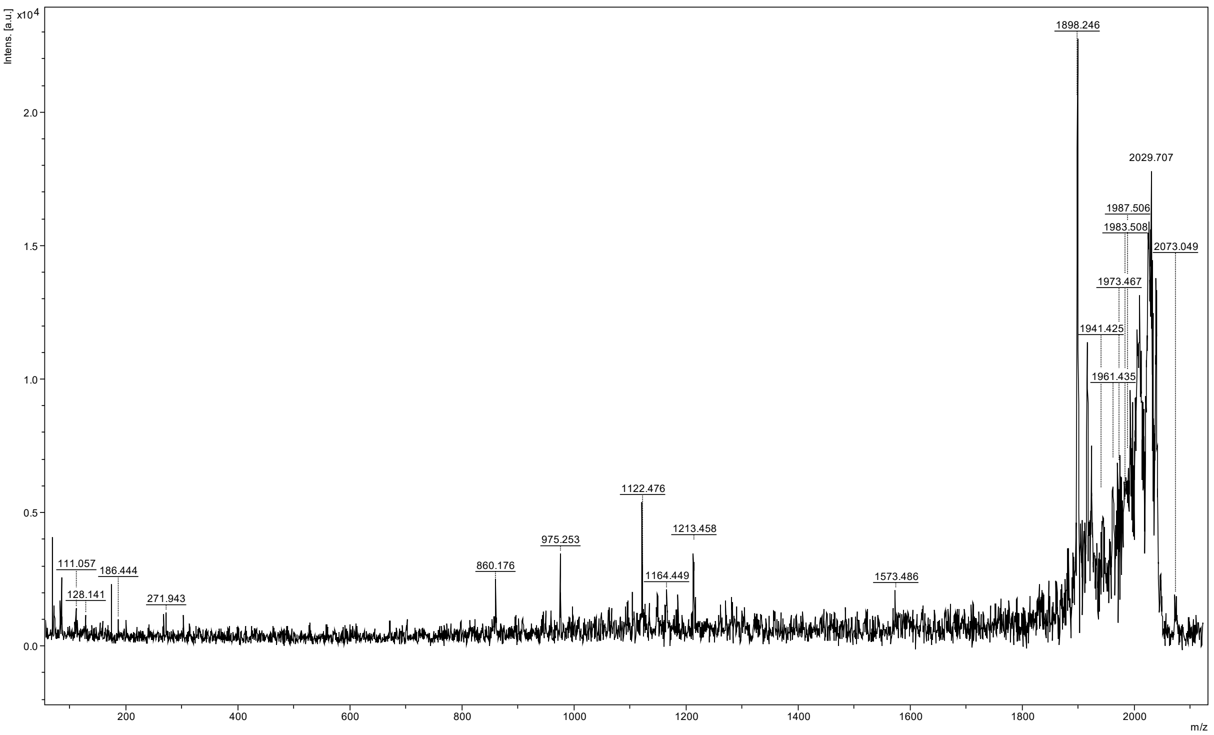

Figure S9. Fragmentation spectra of *m/z* = 2073.0 using RapifleX

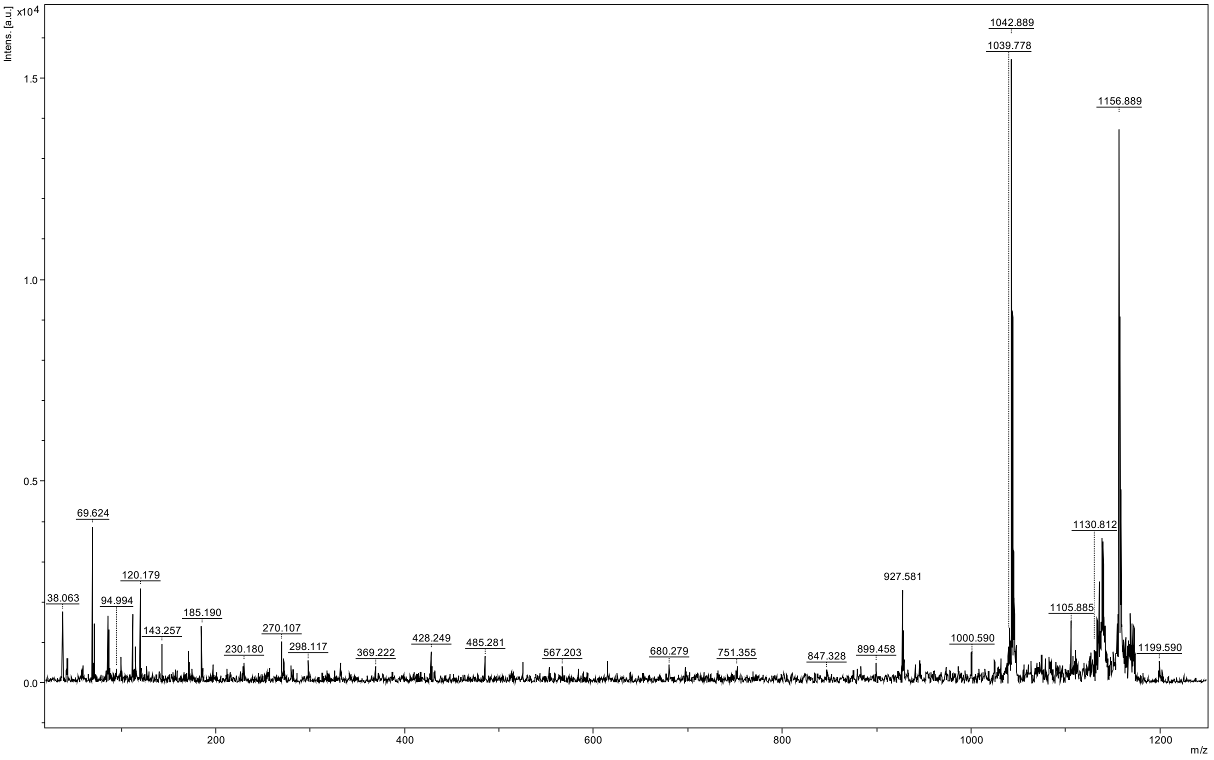

Figure S10. Fragmentation spectra of *m/z* = 1198.7 using RapifleX
